# Prediction of prostate biopsy outcomes at different cut-offs of prostate-specific antigen using machine learning: a multicenter study

**DOI:** 10.1186/s43046-025-00265-3

**Published:** 2025-03-17

**Authors:** Mostafa A. Arafa, Karim H. Farhat, Sherin F. Aly, Farrukh K. Khan, Alaa Mokhtar, Abdulaziz M. Althunayan, Waleed Al-Taweel, Sultan S. Al-Khateeb, Sami Azhari, Danny M. Rabah

**Affiliations:** 1 The Cancer Research Chair, Surgery Department, College of Medicine, King Saud University, Riyadh, Saudi Arabia; 2 Epidemiology Department, High Institute of Public Health, Alexandria University, Alexandria, Egypt; 3 Information Technology Department, Institute of Graduate Studies and Research, Alexandria University, Alexandria, Egypt; 4 Department of Urology, King Faisal Specialist Hospital and Research Center, Riyadh, Saudi Arabia; 5 College of Medicine, Alfaisal University, Riyadh, Saudi Arabia; 6 Surgery Department, College of Medicine, King Saud University, Riyadh, Saudi Arabia

**Keywords:** Machine learning prediction models, Prostate biopsy outcomes, Prostate-specific antigen cut-offs

## Abstract

**Background:**

Machine learning (ML) is a significant area of artificial intelligence, which can improve the accuracy of predictive or diagnostic models for differentiating between prostate biopsy outcomes. This study aims to develop a novel decision-support ML model for classifying patients with biopsy-negative (cancer-free), clinically significant, and non-clinically significant prostate cancer across two prostate-specific antigen (PSA) cut-offs ≤ 10 ng/ml and > 10 ng/ml.

**Methods:**

The data for the current study were retrieved from the records of two main hospitals in Riyadh, Saudi Arabia from July 2018 through July 2024. Six machine learning algorithms were employed, and the dataset was randomly divided into a training set and a validation set at a ratio of 8:2. The following metrics were used as performance indicators across the six algorithms: Accuracy, Precision, Recall, F1-score, and area under the curve. Recent data from the two hospitals was utilized for external validation.

**Results:**

The metrics for Random Forest, Extra Tree, and Decision Tree algorithms showed excellent capability in classifying the outcomes of prostate biopsy for the two PSA cut-offs. However, the metrics for the PSA cut-off > 10 ng/ml are higher than those for PSA ≤ 10 ng/ml. For the three-class classification, the accuracy and area under the curve for the cut-off > 10 ng/ml were 0.96 and 0.99, respectively. While for the cut-off ≤ 10 ng/ml they were 0.92 and 0.94 for Random Forest and 0.94 and 0.95 for the Extra Tree algorithm. The metrics of non-clinically significant and biopsy-negative cases outperformed those of clinically significant cases.

**Conclusion:**

ML models are proving to be effective tools in differentiating between prostate biopsy outcomes, enhancing diagnostic accuracy, and potentially transforming clinical practices in prostate cancer management.

## Introduction

Prostate cancer is the second most common disease to be diagnosed and the fifth most common cause of cancer-related death among men worldwide. It is the most common cancer diagnosed in 112 countries and 48 countries overall. It is crucial to keep in mind that as the population ages and the economy grows, it is expected that the prevalence of prostate cancer will increase [1, 2].

The incidence of prostate cancer in our region is much lower in comparison to European countries, where the reported age-standardized incidence rate (ASIR) in Saudi Arabia was 14.8 [3]. The recent work of Arafa et al. [4] in Saudi Arabia revealed an incidence rate of 0.24%.

Prostate cancer that is aggressive or advanced has a dismal prognosis. It is essential to accurately identify aggressive prostate tumors and investigate clear outcomes, both from an etiological and preventive perspective. Clinically significant prostate cancer (CSPCa) is often described in epidemiological research using many categories of clinical factors, such as stage, Gleason score (GS), and diagnostic value of prostate-specific antigen (PSA) [5].

The criteria for advanced prostate cancer are widely agreed upon, and they include a GS of 3 or higher + 4. By requiring a high diagnostic total PSA to meet these criteria, the sensitivity of diagnosis was increased [5, 6].

Many studies are currently focused on finding advanced or clinically significant PCa predictors for early detection and management. Machine learning (ML), a substantial area of artificial intelligence, can improve the aptitude of predictive or diagnostic models. It is more skilled at handling non-linear correlations than traditional statistical scores. Because of this, ML models have enormous potential for disease detection and prognosis [7, 8]. Machine learning offers several clinical significance in categorizing prostate biopsy outcomes. By improving risk stratification, enabling personalized treatment decisions, enhancing patient outcomes, improving patients’ quality of life by avoiding over-treatment, and optimizing the resources by identifying patients who truly need treatment [9].

Two distinct PSA cut-offs have been commonly used (≤ 10 ng/ml and > 10 ng/ml). It helps guide further investigation; such classification manages patient expectations. Knowing the potential significance of PSA levels helps patients understand the potential implications of their test results, as a PSA less than 10 ng/ml carries a lower risk of prostate cancer, in contrast to a PSA level more than 10 ng/ml [10].

The current work aimed to develop novel decision-supporting ML models for the classification, and prognosis of patients with biopsy-negative (cancer-free), CSPCa, and non-clinically significant prostate cancer (NCSPCa) using real data and to compare these models between two different PSA cut-offs: ≤ 10 ng/ml and > 10 ng/ml, as to clinically stratify patients into two distinct risk groups.

## Methods

The records of the two largest hospitals in Riyadh, Saudi Arabia, King Khalid University Hospital and King Faisal Specialist Hospital, were used to retrieve data from patients who underwent prostate biopsy between July 2018 and July 2024.

Ethical Review Board: Approval was granted by the Ethics Committee of the College of Medicine at King Saud University, Riyadh, Saudi Arabia (No.: 19/0299/IRB). This research is compliant with the guidelines of human studies and was conducted ethically in accordance with the World Medical Association Declaration of Helsinki.

According to the protocols in the two hospitals, all patients with persistently high PSA (≥ 3.5 ng/ml) underwent magnetic resonance imaging (MRI), and those with suspicious findings (PI-RAD 2 to PI-RAD 5). underwent a transrectal ultrasound 12 + X prostate biopsy was performed, and the results were evaluated and recorded. According to the protocols adopted in the two hospitals, the Artemis system (Eigen, Grass Valley, CA, USA) and software were electronically updated with the MRI region of interest. Depending on the size of the lesion, 2–3 cores were collected from the target lesion before a systematic 12-core approach was used for each patient. The following data were registered: age, body mass index (BMI), prostate volume (PV) measured on MRI, total PSA, PSA density, multiparametric MRI (mpMRI) PI-RADS scores, and the results of systematic examination and targeted biopsy. The total PSA value was divided into values less than or equal to 10 ng/ml and more than 10 ng/ml for comparison. The Gleason grading used in the study was changed due to major changes in 2005 [11]. The non-significant PCa was defined as GS < 3 + 4 and the significant PCa as GS ≥ 3 + 4.

### Statistical analysis

Variables with high and significant correlations (*r* > 0.75) were excluded from the analysis to avoid multicollinearity, *P* < 0.05 was considered statistical significance.

### Machine learning approach

The data from the two centers were combined and utilized for ML training and testing procedures.

We have developed a supervised machine learning-based approach to automatically classify benign, CSPCa, and NCSPCa based on various health metrics including age, tPSA, PSA density, prostate volume, and MP-MRI values. The data set was divided into training and test sets in a ratio of 80:20. This is a standard approach to ensure that the performance of the model can be evaluated using unseen data. To train an ML model, we applied the following steps:

- 1- Data preprocessing: feature scaling/standardization was performed on the features. Standardization of features was performed using the standard scaler approach, which standardizes features by removing the mean and scaling to unit variance. This process ensures that each feature contributes equally to the analysis and prevents features with larger ranges from dominating those with smaller ranges.
- 2- Feature selection: for feature selection, the Pearson correlation matrix was calculated to evaluate the linear relationships between the features and the target variable (i.e., data label). This matrix provides information about how strongly each feature correlates with the biopsy result and with each other. By identifying highly correlated features, we can make informed decisions about which features to include or exclude from our predictive model, potentially improving model performance.

We used Python 3.11.5 to create our models. We used six machine learning algorithms namely Linear Regression, XG Boost, Decision Tree, Random Forest, Extra Tree, and Gradient Boosting. We randomly divided the dataset into training sets and validation sets at a ratio of 8:2. ML-based models were optimized to avoid over-fitting, and the accuracy of the algorithm was tested using the tenfold cross-validation method. The following metrics were used as performance indicators for the six algorithms: Accuracy, Precision, Recall, F1-score, and area under the curve (AUC) value (with a 95% confidence interval).

**Fig. 1:**
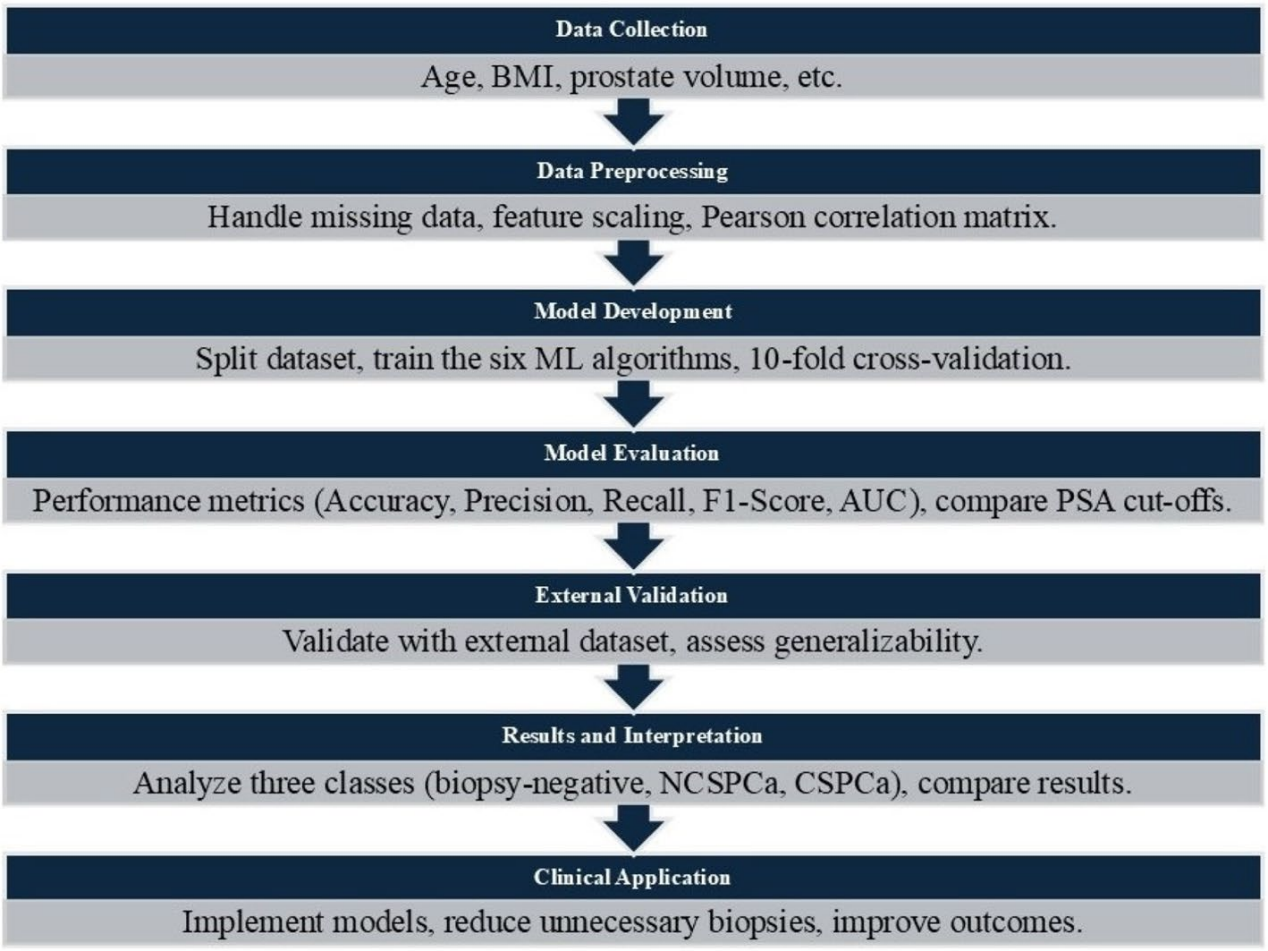
Overall flow diagram of the study

For external validation, data from 205 patients who underwent prostate biopsy between July 2017 and June 2018 were retrieved from the records of the two hospitals, and their information was used. The overall flow diagram of the study is outlined in Fig. 1.

## Results

**Fig. 2:**
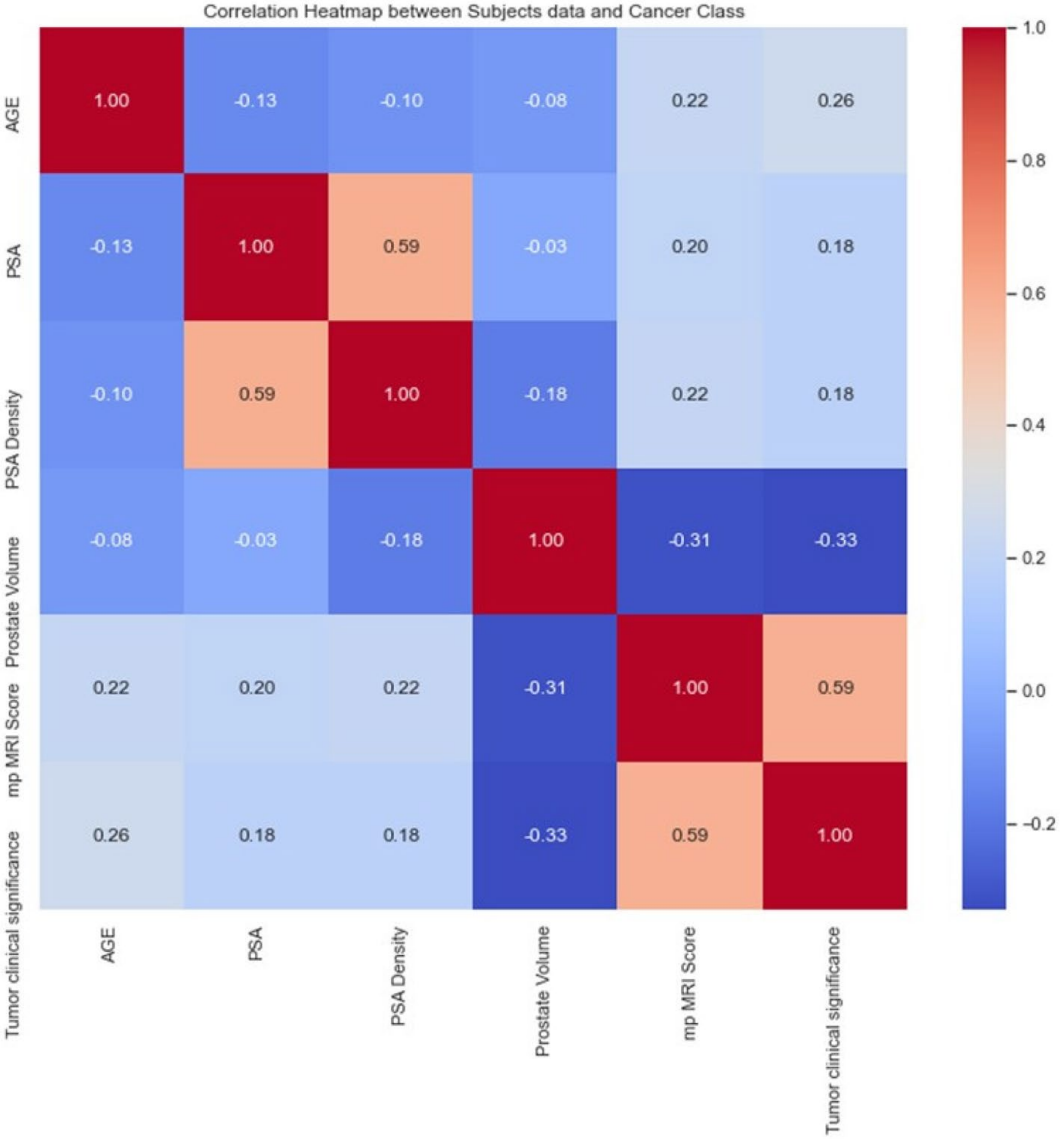
Heat map correlation matrix

The total number of patient records retrieved from the two hospitals during the study period was 997; 28 cases were excluded due to missing data. Ultimately, 969 patient files remained. 577 (59.5%) were cancer-free (biopsy negative), 95 (9.8%) were diagnosed as NCSPCa, and 297 (30.7%) were classified as CSPCa. The mean patients’ age was 65.6 ± 7.1. The patients with a PSA value ≤ 10 ng/ml were 627 (64.7%) patients, while those with a PSA value > 10 ng/ml accounted for 342 (35.3%) of our cohort. The only features that showed a moderate correlation were PSA and PSA density, *r* = 0.59, *P* = 0.04 (Fig. 2).

### Performance indicators for different algorithms

The metrics for the Random Forest (RF), Extra Tree (ET), and Decision Tree (DT) algorithms showed excellent ability to classify between the prostate biopsy results compared to other algorithms for the two PSA cut-offs; nevertheless, the measured values for the PSA cut-off > 10 ng/ml are higher than those for PSA ≤ 10 ng/ml. The accuracy and AUC for the cut-off > 10 ng/ml were 0.96 and 0.99, respectively, while those for the cut-off ≤ 10 ng/ml for Random Forest were 0.92 and 0.94 and for the Extra Tree algorithm were 0.94 and 0.95, Tables 1 and 2.

**Table 1:** Performance of the models for cases with a PSA > 10

Classifier	Accuracy	Precision	Recall	F1-Score	AUC [95% CI]
Linear regression	0.72	0.691	0.72	0.704	0.64 [0.60, 0.84]
XGBoost	0.92	0.92	0.92	0.92	0.977 [0.92, 0.99]
Decision Tree	**0.96**	**0.963**	**0.96**	**0.96**	**0.969 [0.92, 0.99]**
Random Forest	**0.96**	**0.964**	**0.96**	**0.96**	**0.994 [0.92, 1.00]**
Extra Tree	**0.96**	**0.976**	**0.96**	**0.964**	**0.998 [0.92, 1.00]**
Gradient boosting	0.96	0.964	0.96	0.96	0.984 [0.92, 1.00]

**Table 2:** Performance of the models for cases with a PSA < = 10

Classifier	Accuracy	Precision	Recall	F1-Score	AUC [95% CI]
Linear regression	0.775	0.68	0.775	0.724	0.845 [0.80, 0.97]
XGBoost	0.922	0.921	0.922	0.919	0.89 [0.82, 0.97]
Decision Tree	**0.961**	**0.963**	**0.961**	**0.96**	**0.925 [0.90, 0.98]**
Random Forest	**0.922**	**0.921**	**0.922**	**0.919**	**0.944 [0.89, 0.98]**
Extra Tree	**0.941**	**0.946**	**0.941**	**0.938**	**0.957 [0.92, 1.00]**
Gradient boosting	0.892	0.888	0.892	0.888	0.934 [0.92, 0.99]

### *For cases with PSA* > *10 ng/mL*

**Fig. 3:**
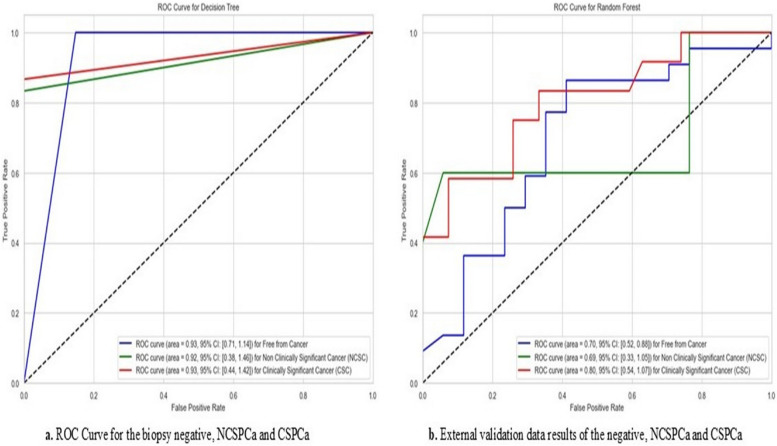
ROC curve for cases with PSA > 10 ng/ml

The ROC curve in Fig. 3a showed excellent discriminatory power for NCSPCa, followed by CSPCa and biopsy-negative cases (AUC was 1, 0.98, and 0.97, respectively). For external validation cases in Fig. 3b, the AUC was 0.90 for cancer-free cases and 0.89 for CSPCa cases. None of the cases were classified as NCSPCa cases in this category.

### *For cases with PSA* ≤ *10 ng/ml*

**Fig. 4:**
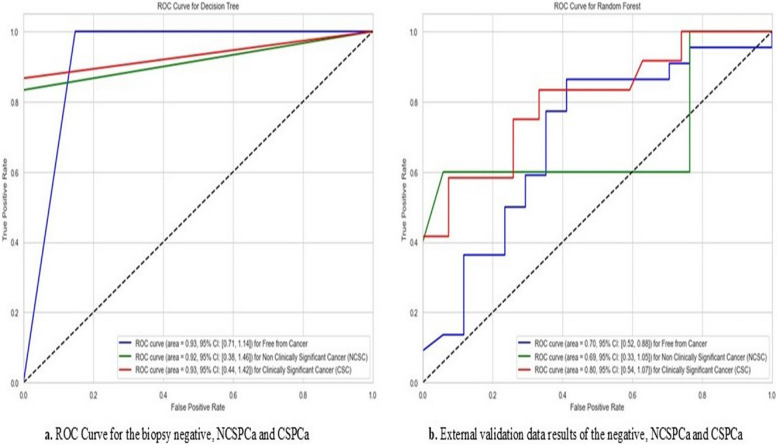
ROC curve for cases with a PSA < = 10

The classification performance of the machine learning algorithms for PSA ≤ 10 ng/ml is very high for the three outcomes, with the AUC of the ROC curve ranging from 0.92 to 0.093. Nevertheless, it is lower than that for the PSA cut-off > 10 mb/ml (Fig. 4a).

The external validation data showed good discriminatory power for CSPCa (AUC = 0.8), while this was the case for biopsy-negative cases and NCSPCa was moderate (AUC was 0.7 and 0.69, respectively) (Fig. 4b).

The metrics of NCSPCa and biopsy-negative cases exceeded those of CSPCa for both groups with a PSA > 10 and PSA < = 10 ng/ml, as shown in Table 3.

**Table 3:** Classification report

	PSA > 10 ng/ml				PSA < = 10 ng/ml				
	Precision	Recall	F1-score	Accuracy	Precision	Recall	F1-score	Accuracy	Precision
Biopsy negative	1.00	0.93	0.96	0.98	0.95	1.00	0.97	0.98	0.95
NCSPCa	1.00	1.00	1.00	0.97	1.00	0.83	0.91	0.96	1.00
CSPCa	0.91	1.00	0.95	0.96	1.00	0.87	0.93	0.96	1.00

## Discussion

In the last decade, PSA has been widely used as the most valuable diagnostic and prognostic marker for PCa [12]. However, it has been shown that less than 30% of men with PSA values in the gray zone (4–10 ng/ml) have pathologically confirmed PCa, suggesting that a large proportion of patients have undergone unnecessary biopsies and were overdiagnosed [13].

All machine learning models used in the current study (except linear regression) demonstrated excellent performance in discriminating between prostate biopsy results, particularly in patients with PSA > 10 ng/ml, their internal and external validity was excellent, as evidenced by the corresponding performance indicators and the AUC. We included tPSA, PSA density, age, PV, and mpMRI score for building the ML models. The model used performs very well in all classes, with particularly high precision and recall for NCSPCa and biopsy-free cases of “non-clinically significant cancer”. For CSPCa, the precision is slightly lower at 91%, suggesting that there are some false positives, but the recall is perfect at 100%, meaning that all true positive cases have been identified. The high accuracy (96%) and strong F1-scores indicate that the model is reliable and performs well in distinguishing between the three classes.

As for cases with PSA < = 10 ng/mL, the model performs exceptionally well on biopsy-negative cases with perfect recall and very high precision, indicating very few false positives and no false negatives. For NCSPCa, the precision is perfect, but the recall is slightly lower at 0.83, indicating some false negatives. The F1-score of 0.91 shows a strong overall performance. An accuracy of 0.96 indicates that the model correctly classifies 96% of the instances in the test set, demonstrating high overall performance.

In a study including patients with tPSA < 10 ng/ml, a PSA-based machine learning model was constructed based on a dense neural network with an AUC of 0.72, which was improved compared to PSA alone, age, fPSA, and f/tPSA alone [14]. In the same context, the study of Peter Ka-Fung Chiu et al. included four variables, PSA, DRE, PV, and transrectal ultrasound findings, were included, and Support Vector Machines (SVM), LR, and RF models were constructed. All models were shown to have better predictions for PCa and clinically significant PCa than PSA and PSAD alone [15].

The ML models proposed in various studies achieved high performance in predicting negative prostate biopsy results for patients with negative mpMRI using both radiomics and clinical features. In the first case, the AUC showed a very high discrimination ability (0.95) compared to the second (0.79) [16, 17]. However, the two models achieved high performance with a negative predictive value of over 95%.

AI models have shown significant effectiveness in distinguishing between CSPCa and NCSPCa [18, 19]. This differentiation is critical to improving patient management and reducing unnecessary biopsies. AI models, particularly those using deep learning techniques on mpMRI images, have shown high accuracy in predicting CSPCa. For example, one study reported an accuracy of 94.3% in CSPCa patients compared to 77.6% in NCSPCa patients, highlighting a significant ability to effectively detect clinically significant cases [18].

The study by Yu S. et al. reported that machine learning models, particularly SVM and RF, achieved an AUC of 0.903 for PCa and 0.925 for clinically significant prostate cancer CSPCa. These models outperformed conventional methods and demonstrated their potential for clinical applications, reducing the number of unnecessary biopsies by up to 57% with a sensitivity of 95% [20].

The current study showed promising and outstanding results, such as those reported by Chen S. et al. who reported that all five models had good calibration in the training data set, the RF, DT, and multivariate LR models showed better discrimination with AUCs of 1.0, 0.922, and 0.91 [21]. However, ongoing validation in diverse and larger cohorts is essential to confirm the clinical applicability of these AI models prior to widespread implementation in clinical practice.

ML has shown clinical significance for improved risk stratification; and accurate CSPCa detection which allows for more targeted biopsies and reduces the risk of unnecessary procedures in low-risk patients. Identification of NCSPCa: it helps avoid overtreatment, and prediction of biopsy negativity, which can prevent unnecessary biopsies in patients with a low risk of cancer, reducing patient anxiety and healthcare costs.

Machine learning models can be applied in real-time by integrating models into the Clinical Decision Support System can provide real-time recommendations to clinicians during patient consultations, suggesting when to consider PSA levels as indicative of potential cancer. It can also analyze electronic health records data to ensign patients with rising PSA levels that may warrant further investigation. For these applications to be effective, the machine learning models must be trained on high-quality, diverse datasets, validated for accuracy and reliability, and integrated into the decision-making process of healthcare providers.

The major limitation of the present study is that it was conducted retrospectively, and some variables were not included in the models due to missing data from the records such as lesion size and free PSA. Another possible limitation is the lack of radiological features extracted directly from MRI images. In the future, implementing our algorithm with radiomics features may help further improve its performance as they have the potential to significantly improve the diagnosis and treatment of prostate cancer. Ultimately, the patients with negative mpMRI were not evaluated as a separate cohort.

## Conclusion

Machine learning has the potential to significantly improve the categorization of prostate biopsy outcomes into CSPCa, NCSsPCa, and biopsy negative. The DT, RF, and ET classifiers show strong performance across all metrics, particularly for cases with PSA > 10 ng/mL, with particularly high precision and recall for the cancer-free and clinically significant cancer classes. The slightly lower recall for the non-clinically significant cancer class suggests room for improvement, but the overall performance is very good. Machine learning models have demonstrated high accuracy and clinical utility in predicting biopsy results in prostate cancer. With AUC values often exceeding 0.95 and significant improvements over traditional methods, these models represent a valuable tool in the diagnostic process and aid in decision-making for prostate cancer treatment. Ongoing research and clinical trials are essential to further explore the potential of machine learning in prostate cancer diagnosis.

## Data Availability

The dataset generated during and/or analyzed during the current study are available from the corresponding author on reasonable request.
